# Prenatally Ruptured Patent Urachus: A Case Report and Review of Literature

**DOI:** 10.3390/medicina58111621

**Published:** 2022-11-10

**Authors:** Ji-Young Kwon, Seung-Yeon Pyeon

**Affiliations:** 1 Department of Medicine, Graduate School, Kyung Hee University, 26 Kyungheedau-ro, Dongdaemun-gu, Seoul 02447, Korea; 2 Department of Obstetrics and Gynecology, Kyung Hee University Hospital at Gangdong, 892 Dongnam-ro, Gangdong-gu, Seoul 05278, Korea

**Keywords:** patent urachus, urachal cyst, urachus

## Abstract

Patent urachus is a type of urachal anomaly in which the urachus does not tail off but remains connected to the bladder in the umbilicus. The prevalence of patent urachus is very low. Herein, we report a case of patent urachus ruptured and exposed to amniotic fluid in utero. In this case, the size decreased after the second trimester, which was thought to be due to rupture in utero. After delivery, patent urachus was confirmed by inserting a foley catheter, which runs through a ruptured cyst on umbilical cord insertion. The day after delivery, the neonate underwent surgical excision of the urachal cyst and closing umbilicus. The mechanism of patent urachus rupture is unknown. As the fetus matures, it is thought that the higher intravesical pressure may affect the rupture of the cyst. Patent urachus could be ruptured in the uterus spontaneously, and surgical correction is needed. Therefore, prenatal differential diagnosis is important.

## 1. Introduction

Patent urachus, a remnant of the embryonic allantois, is defined as an abnormality of the umbilicus connecting with the bladder. Patent urachus is rare as it is reported in 1 to 2 or 2.5 per 1,000,000 live births [1]. Prenatal sonographic findings include increased thickness of the umbilicus, and can be observed as an extra-abdominal cystic mass. It appears as a tubular fluid-filled structure between the anterosuperior aspect of the bladder and umbilicus, with a hypoechoic wall and anechoic lumen. It could reduce by drainage of urine into the bladder through patent urachus [2]. However, there are a few cases of patent urachus prenatally ruptured in the uterus, which seemed to be resolved in the second and third trimesters. Herein, we report a case of a patent urachus found at the gestational age of 13 + 4 weeks and disappeared in the third trimester, and is thought to have ruptured in utero.

## 2. Case Presentation

**Figure 1:**
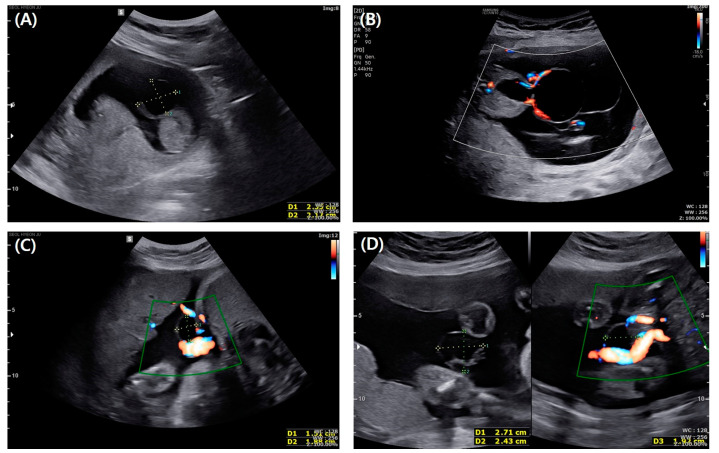
(**A**) Sonographic finding of the urachal cyst at the gestational age of 13 + 4 weeks shows a 2.4 cm well–defined oval–shaped anechoic cyst located at the proximal part of the umbilical cord. (**B**) Sonographic finding of the urachal cyst at the gestational age of 17 + 4 weeks shows the size of the cyst increased to 5.4 cm, with a newly appeared 3 cm–sized cyst inside the initial cyst. (**C**) Sonographic finding of the urachal cyst at the gestational age of 25 weeks shows the size of the cyst has decreased to 2.7 cm. (**D**) Sonographic finding of the urachal cyst at the gestational age of 32 weeks shows it became 1.8 cm in size.

**Figure 2:**
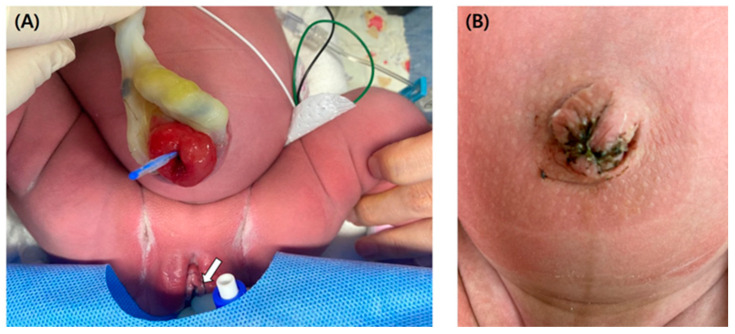
(**A**) Picture of a Foley catheter passing through a ruptured cyst at umbilical cord insertion. (The white arrow indicates the beginning of the Foley catheter). (**B**) Picture of umbilicus of neonate after surgery.

A 35-year-old woman, gravida 1, para 1, was referred to our hospital at the gestational age of 13 + 4 weeks with suspicion of omphalocele. She had previous dichorionic diamniotic twin fetuses born by cesarean section at the gestational age of 35 weeks and medical history of total thyroidectomy due to thyroid cancer. Her current medications when she was referred to our hospital were synthyroxine and antiemetic drugs. At her first visit to our hospital, at the gestational age of 13 + 4 weeks, the ultrasound showed a 2.4 cm well-defined oval-shaped anechoic cyst at the proximal part of the umbilical cord. The ultrasound found a connection between the cyst and bladder, and we observed no hernia of the intestine from the abdominal wall. Umbilical vessels were also intact (Figure 1A). Therefore, we highly suspected an umbilical cyst or urogenital anomaly. However, unsure of the possibility of omphalocele, we recommended genetic testing. At the gestational age of 15 + 3 weeks, amniocentesis was performed. The results of karyotyping and chromosomal microarray were normal. At the gestational age of 17 + 4 weeks, the size of the cyst had increased to 5.4 cm with a newly appeared 3 cm sized cyst inside the initial cyst and no other associated anomaly (Figure 1B). At the gestational age of 25 weeks, the size of the cyst decreased to 2.7 cm (Figure 1C), and at 32 weeks, it became 1.8 cm. At term, the cyst was too small to find on ultrasound examination (Figure 1D). The bladder was always visible from the first to the third trimester. She was delivered by cesarean section at the gestational age of 39 + 4 weeks due to prior cesarean section history. After delivery, a ruptured urachal cyst was observed at the neonate’s umbilicus. Patent urachus was confirmed by inserting a foley catheter, which runs through a ruptured cyst on umbilical cord insertion (Figure 2A). The day after delivery, the neonate underwent surgical excision of the urachal cyst and closing umbilicus (Figure 2B). The baby recovered well and was discharged without any other complications.

## 3. Discussion

The urachus, a tubular structure connecting the cloaca and allantois, which later develop into the bladder and umbilicus, respectively, becomes obliterated later in normal embryology. When this normal embryologic tissue fails to obliterate, a connection between the bladder and umbilicus remains. These anomalies include patent urachus (47%), urachal cyst (30%), umbilical-urachal sinus (18%), and vesico-urachal diverticulum (3%), classified by the level of persistence of the embryonic urachal remnants between the bladder and umbilicus [3,4]. In our case, patent urachus was ruptured in the uterus and was not observed in the third trimester. We found some cases in pubmed.gov with keywords of “urachal cyst”, “ruptured”, and “in utero” written in English. As a result, 12 articles were found. Among the results, we reviewed six articles (including 10 cases) similar to ours. A brief review of these cases is presented in Table 1 [1,5,6,7,8,9]. In one case, omphalocele was present, but there was no genetic anomaly. Another case had a genetic anomaly. Among the reviewed cases, the cysts disappeared at 26–30 weeks and were thought to be spontaneous ruptures. All cases had an operation within a few birthdays, and the prognosis was good.

The mechanism of patent urachus rupture is unknown. One plausible hypothesis related to patent urachus is increased pressure within the bladder, such as in obstructive uropathy. Pal et al. reported a case of a male fetus with an allantoic cyst and cordee with hypospadias and meatal obstruction. In the case report, a small defect of the allantoic cyst made possible to reduce the pressure of the bladder, thus, minimizing the morbidity of urethral obstruction such as a kidney injury [10]. Bureau et al. introduced a case of a fetus with patent urachus and posterior urethral valves [11]. The authors of the above two cases suggested that meatal obstruction could be a plausible cause for patent urachus. If a urachus exist, increased pressure within the bladder possibly results in a urachus-remained patent. However, the coexistence of urachal anomaly and obstructive uropathy is very rare. Therefore, as is in our case, obstructive uropathy was not present. As the fetus matures, it is thought that the higher intravesical pressure may impact the rupture of the cyst [8]. When the urachal cyst ruptures in the uterus, a part of the bladder is exposed to the amniotic fluid through the patent urachus. In this situation, bladder exstrophy should be excluded. Unlike urachal anomalies, bladder exstrophy, a rare congenital anomaly, results from a defect of middling mesodermal fusion and cloacal membrane rupture, accompanied by abdominal wall defect and cloacal exstrophy, which could co-occur in severe cases. Bladder and cloacal exstrophies have a poorer prognosis than patent urachus. The point of differential diagnosis is whether a normal bladder is visible and whether the infraumbilical abdominal wall is intact [12]. 

The cyst in front of the fetal abdomen should be distinguished from the umbilical cord cyst and omphalocele. Based on histopathological findings, an umbilical cord cyst is classified into a true cyst or a pseudocyst [8]. True cysts arise from remnants of allantois and can be associated with patent urachus, omphalocele, and hydronephrosis. A pseudocyst, more common than a true cyst, is caused by local edema or atrophy of cord epithelium and is associated with chromosomal anomalies [8]. Pseudocysts are usually smaller and can be located anywhere in the umbilicus. The prenatal differential diagnosis of a pseudocyst, true cyst, and patent urachus is difficult. Omphalocele, a hernia in which abdominal organs protrude into the umbilical cord, could be ruled out by bowel contents within the channel [1]. 

## 4. Conclusions

Patent urachus could rupture in the uterus spontaneously, requiring surgical correction. In patent urachus, the accompanying anomaly is rare, and the prognosis is good. Therefore, prenatal differential diagnosis is important. Regularly targeted ultrasonography is crucial when an umbilical cord cyst is diagnosed in the first trimester, even if the cyst disappears.

## Figures and Tables

**Table 1:** A brief review of similar cases.

The Author of the Previous Case Report	GA at Detection	GA at Disappearance	Performance of Genetic Evaluation (Result)	GA at Delivery	Mode of Delivery	Accompanying Anomaly	Prenatal Sonographic Finding	Postnatal Finding
Yoo et al. [5]	16	29	Yes (NL)	38	CS	SUA omphalocele	A large cyst in the umbilical cord which was connected to the fetal bladder and seemed like a dumb-bell	Omphalocele and exstrophy of the urachus
Riddell et al. [6]	20	30	No	37	VD	Vesico-ureteral reflux (grade 3)	Hourglass appearance of the cyst with communication between the dome of the bladder and the allantois in the umbilical cord	Prolapsed bladder
Svigos et al. [7]	12	Not indicated	Yes (NL)	35	CS	Not indicated	Hypoechoic area on the anterior abdominal wall	A patent urachus
Trong et al. [1]	13	27	Yes (NL)	38	CS	SUA	Cystic mass from the root of the umbilical cord connected to the urinary bladder.	Ruptured allantoic cyst with patent urachus in single umbilical artery
Umeda et al. [8] ^£^	15–27	26–35	Yes (22q11.2 deletion syndrome)	35–39	Not indicated	TOF	Cyst in the umbilical cord.	Patent urachus and urachal cyst
Srisupunditet al. [9]	22	28	Yes (NL)	38	VD	None	Extra-abdominal cystic mass at the base of umbilical cord	Rupture of the patent urachus/urachal cyst with bladder prolapse

GA, gestational age; NL, normal; VD, vaginal delivery; CS, cesarean section; SUA, single uterine artery; TOF, tetralogy of Fallot. ^£^ This article included 5 cases.

## Data Availability

Not applicable.

## References

[1] Thach T.T., Quan V.D., Nghi T.D., Anh N.H., Hung L.P., Luan N.T., Long N.P. (2015). Case Report: Pre-and postnatal management of an allantoic cyst with patent urachus and single umbilical artery. F1000Research.

[2] Rasteiro C., Ramalho C., Loureiro T., Pereira J., Matias A. (2012). Bladder emptying into an umbilical cord cyst: Prenatal sonographic sign of allantoic cyst with patent urachus. Ultrasound Obstet. Gynecol..

[3] Awwad J., Azar G., Soubra M. (1994). Sonographic diagnosis of a urachal cyst in utero. Acta Obstet. Gynecol. Scand..

[4] Buddha S., Menias C.O., Katabathina V.S. (2019). Imaging of urachal anomalies. Abdom. Radiol..

[5] Yoo S.-J., Lee Y.-H., Ryu H.M., Joo M.S., Cheon C.K., Park K.W. (1997). Unusual fate of vesicoallantoic cyst with non-visualization of fetal urinary bladder in a case of patent urachus. Ultrasound Obstet. Gynecol..

[6] Riddell J.V.B., Houle A.-M., Franc-Guimond J., Barrieras D. (2015). Prenatal vesico-allantoic cyst outcome—A spectrum from patent urachus to bladder exstrophy. Prenat. Diagn..

[7] Svigos J., Khurana S., Munt C., Sinhal S., Bernardo J. (2013). Presentation of an umbilical cord cyst with a surprising jet: A case report of a patent urachus. F1000Research.

[8] Umeda S., Usui N., Kanagawa T., Yamamichi T., Nara K., Ueno T., Owari M., Uehara S., Oue T., Kimura T. (2016). Prenatal and Postnatal Clinical Course of an Urachus Identified as an Allantoic Cyst in the Umbilical Cord. Eur. J. Pediatr. Surg..

[9] Srisupundit K., Mahawong P., Charoenratana C., Tongsong T. (2018). Prolapsed bladder following rupture of patent urachal cyst, mimicking bladder exstrophy: A case report and literature review. J. Med. Ultrason..

[10] Pal K., Ashri H., Al-Ghazal F.A. (2008). Allantoic cyst and patent urachus. Indian J. Pediatr..

[11] Bureau M., Bolduc S. (2011). Allatoic cysts and posterior urethral valves: A case report. Ultrasound Obs. Gynecol..

[12] Tong S.-Y., Lee J.-E., Kim S.-R., Lee S.-K. (2007). Umbilical cord cyst: A prenatal clue to bladder exstrophy. Prenat. Diagn..

